# Wear Mechanisms of the Working Surface of Gears after Scuffing Tests

**DOI:** 10.3390/ma17143552

**Published:** 2024-07-18

**Authors:** Edyta Osuch-Słomka, Remigiusz Michalczewski, Anita Mańkowska-Snopczyńska, Marek Kalbarczyk, Andrzej N. Wieczorek, Emilia Skołek

**Affiliations:** 1 Tribology Centre, Łukasiewicz Research Network-Institute for Sustainable Technologies (Ł-ITEE), ul. Pulaskiego 6/10, 26-600 Radom, Poland; remigiusz.michalczewski@itee.lukasiewicz.gov.pl (R.M.); anita.mankowska@itee.lukasiewicz.gov.pl (A.M.-S.); marek.kalbarczyk@itee.lukasiewicz.gov.pl (M.K.); 2 Faculty of Mining, Safety Engineering and Industrial Automation, Silesian University of Technology, ul. Akademicka 2, 44-100 Gliwice, Poland; 3 Faculty of Materials Science and Engineering, Warsaw University of Technology, ul. Wołoska 141, 02-507 Warsaw, Poland; emilia.skolek@pw.edu.pl

**Keywords:** gear teeth, surface analysis, wear mechanisms, coating, scuffing

## Abstract

Identification of changes occurring on the working surface of lubricated gears using analytical equipment, e.g., an FE-SEM scanning electron microscope with an EDS microanalyzer, a WLI interferometric microscope, or a GDEOS optical discharge spectrometer, enables the characterisation of wear mechanisms of this surface. Definition of the phenomena occurring on the surface of tribo-couples after scuffing tests enables a comparative analysis of scuffing resistance and surface properties of the micro- and nanostructure, and elemental composition of the tested gears. Recognition and analysis of the wear mechanisms occurring on the working surface of gears will reduce the risk of damage and losses resulting from the need for maintenance and repair. The study concerned the working surfaces of gears made of 17HNM and 35HGSA steels on which a W-DLC/CrN coating was deposited. Shell Omala S4 GX 320 commercial industrial oil with a synthetic PAO (polyalphaolefin) base was selected for the lubrication of the gears. Tribological tests employed an FZG gear scuffing under severe conditions test method and they were carried out on a T-12U test rig for cylindrical gear analysis.

## 1. Introduction

Manufacturers of gears intended to work under extreme conditions (e.g., mining conveyors) and manufacturers of machines used in agriculture, construction, or forestry require machine elements to be durable and reliable. This means that they must be made from materials that meet stringent requirements and minimise the risk of machine failures, thereby reducing operating costs.

Researchers strive to demonstrate the possibility of increasing the scuffing resistance of lubricated tribosystems through the modification of the working surface of gears [1,2,3,4,5,6,7]. 

The development of new materials and coatings goes hand in hand with the need for the study of tribological properties of tribosystems (e.g., gears), originally intended to verify the quality of newly developed gear oils. The most popular gear testing system is the one developed at the Gear Research Centre (Forschungsstelle fur Zahnrader und Getriebebau, FZG) of the Technical University of Munich [8,9,10]. FZG methods allow the examination of lubricants and the analysis of the impact of surface modification technologies (e.g., coating deposition) on gear durability. For many years, these methods have been successfully used at the Łukasiewicz Research Network—ITEE, Radom, Poland [11,12,13,14]. The most dangerous forms of gear wear include surface scuffing, fatigue (pitting, micropitting), and tooth breakage, so recognising and analysing the phenomena occurring on the working surface of gears is an essential part of the research. It allows the characterisation of wear mechanisms occurring on the working surface of gears and enables differentiation of materials used for heavily loaded gears [15,16,17,18,19,20,21,22,23]. The article identifies changes occurring on the working surface of the teeth of cylindrical gears made of 17HNM and 35HGS steel on which a W-DLC/CrN coating was deposited, after seizure tests. For this purpose, microscopic techniques including the use of a scanning electron microscope, white light interferometer microscope, atomic force microscope, and optical microscope were used. The research aims to extend cylindrical gears’ operating time in the mining industry.

## 2. Materials and Methods

For the purpose of the tests, gears made of the 17HNM steel in the classic carburized and hardened technology with a hardness of 733 ± 14 HV and gears made of nanobainitized 35HGSA steel with a hardness of 671 ± 20 HV were used. The nanobainitization process involves heat treatment of steel, including heating to the austenite phase, followed by austempering (bainitic hardening). Unlike ordinary hardening, during the cooling phase of this process, the steel is held at a temperature higher than the martensite formation temperature, but not exceeding 350 °C, until the austenite transforms into bainite [24,25,26,27,28].

A Balinit C Star commercial coating made of a W-DLC/CrN material reinforced with a CrN transition layer was deposited on the gear surface, allowing heavy load transmission. Coating was deposited on the gear surface by Oerlikon Balzers Coating Poland using a PACVD technology. The aim was to increase scuffing resistance by at least 10%, compared to surfaces made using an existing technology. 

**Figure 1:**
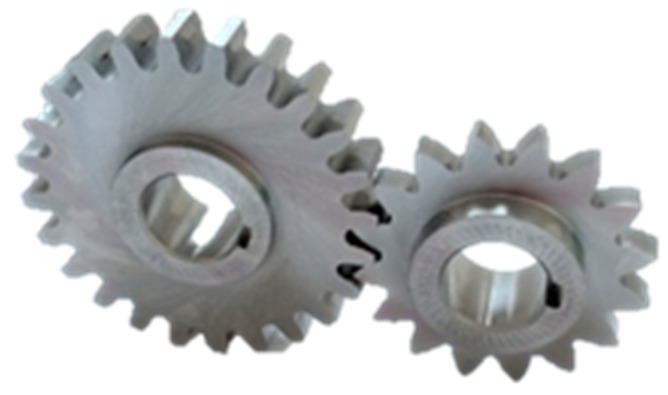
A10 scuffing test gears.

A10 FZG test gears with a small gear (pinion) tooth width of 10 mm and a large gear tooth width of 20 mm were used. The working surface of gear teeth was ground longitudinally (Figure 1). The gears were manufactured by Patentus Poland.

The physical and mechanical properties of the coating are presented in Table 1. Coating adhesion was evaluated using scratch adhesion tests conducted with CSM Instruments Revetest equipment (Peseux, Switzeland) with a standard Rockwell C diamond indenter, a table speed of 10 mm/min, a load increase rate of 100 N/min, and a scratch length of 10 mm. The surface roughness of samples was measured using a white light interferometry microscope (WLIM) by Taylor Hobson. 

The test used a commercial industrial gear oil—Shell Omala S4 GX 320 (London, UK)—with a synthetic PAO (polyalphaolefin) base and AGMA 6/6EP viscosity class.

**Figure 2:**
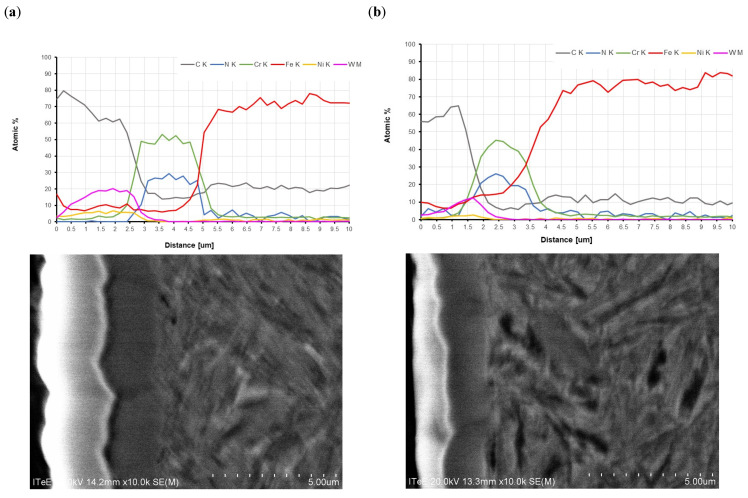
SEM microscope images: (**a**) 17HNM steel/W-DLC/CrN coating, (**b**) 35HGSA steel/W-DLC/CrN coating and GDOES depth profile of W/DLC/CrN coating.

Figure 2 presents the results of the analysis of elemental composition of the coating deposited on the 17HNM and 35HGSA steel substrates using a Jobin Yvon Horiba optical glow discharge emission spectrometer (GDOES). During the analysis of the elements’ distribution curves as the function of distance from the surface, the coating thickness was determined at the intersection of the curves.

The W-DLC/CrN coating consists of three layers. A CrN layer is deposited on the steel substrate; it is an interlayer improving the adhesion of WC to the steel substrate. The hard tungsten carbide (WC) is responsible for transferring heavy loads and when the outer layer is worn out, it protects the substrate from abrasion. The outer layer consists of a WC/C multilayer, including a WC layer and a DLC layer.

### Scuffing Tests

Tribological tests employing the scuffing method under severe conditions were conducted using the A10/166R/90 method described in the following standards: ISO 14635-2 [29], CEC L-84-02 [30], and FVA No. 243, June 2000 [8]. 

**Figure 3:**
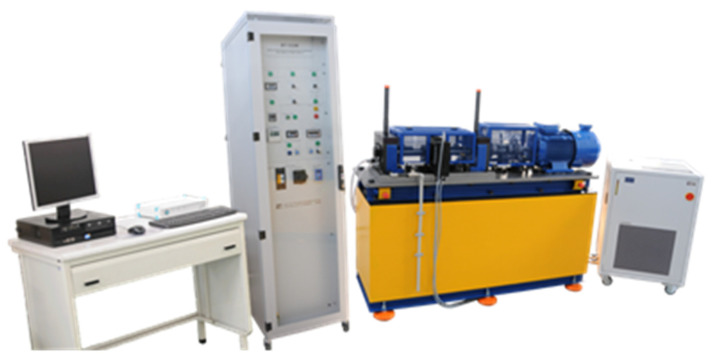
T-12U face-to-face gear test rig.

The tests were carried out on a T-12UF test rig developed at the Łukasiewicz Institute for Sustainable Technologies in Radom (Figure 3), at a constant rotational speed, gradually increasing load, and an initial oil temperature identical at the start of each test run until reaching the failure load stage (FLS).

Test conditions:
-engine rotational speed3000 rpm-rolling speed16.6 m/s-min. and max. load stagefrom 1 to 10-torque loadfrom 3 to 373 Nm, changed gradually-Hertz pressure on the small gear (pinion)from 0.2 to 2.2 GPa-initial test oil temperature90 °C (not stabilised during tests)-type of lubricationimmersion

The tests are conducted until the scuffing criterion is met, i.e., the failure load stage (FLS) causes tooth damage on the small gear across an area greater than 100 mm^2^ or the maximum load stage 10 is reached. Where load stage 10 is reached without scuffing, the large gear should be disassembled and weighed. The result is considered valid if the weight loss of the large gear does not exceed 20 mg. If the scuffing criterion is met, the result is valid regardless of the wear of the large gear (in this case, weighing can be omitted).

## 3. Results

Tribological tests conducted using the gear scuffing test method under severe conditions allowed for the observation of wear propagation at a gradually increasing load. The working surface of the pinion tooth was analysed to identify the wear mechanisms present (Table 2).

The primary parameter defining scuffing resistance is the failure load stage (FLS). Table 3 presents the results of the visual assessment of surface damage on the small gear at individual load stages, starting from load stage 4 to load stage 10. The wear surface area, denoted by parameter A_p_ [mm^2^], was also determined from the working surface of the pinion teeth. 

The lowest wear surface area value of A_p_ = 7.0 mm^2^ was observed for the 35HGSA steel with a W-DLC/CrN coating, whose working surface of the teeth was characterised mainly by scratches. On the other hand, the highest wear surface area value of A_p_ = 144.0 mm^2^ was observed for the 35HGSA steel. The resulting damage of the pinion surface covered an area exceeding 100 mm^2^, which confirms scuffing. Adverse forms of wear, such as micropitting, were observed using SEM. In the case of the 17HNM steel, the surface area reached A_p_ = 23.0 mm^2^, while for the 17HNM steel with a W-DLC/CrN coating, it reached A_p_ = 52.0 mm^2^.

Table 4 presents the condition of the working surface of the pinion teeth at a maximum load level of 10, as observed using a Nikon MM-40 optical microscope (Tokyo, Japan). The analysis of the surface of the pinion teeth in an area of 2.5 mm × 2.5 mm allowed the identification of the most common damage taking place. In the case of teeth made of the 17HNM steel, scratches predominated; at load level 6 they were mostly replaced by grooves. Grooves and characteristic shiny (polished) areas caused by coating wear, occurring in the lower part of the tooth, were observed on the surface made of the 17HNM steel with a W-DLC/CrN coating. The surface made of the 35HGSA steel with a W-DLC/CrN coating was characterised by the presence of scratches and grooves (the latter occurring at load level 9). In the case of the 35HGSA steel, where more than 50% of the working surface of the teeth was destroyed, scuffing of the working surface of the pinion was observed.

**Figure 4:**
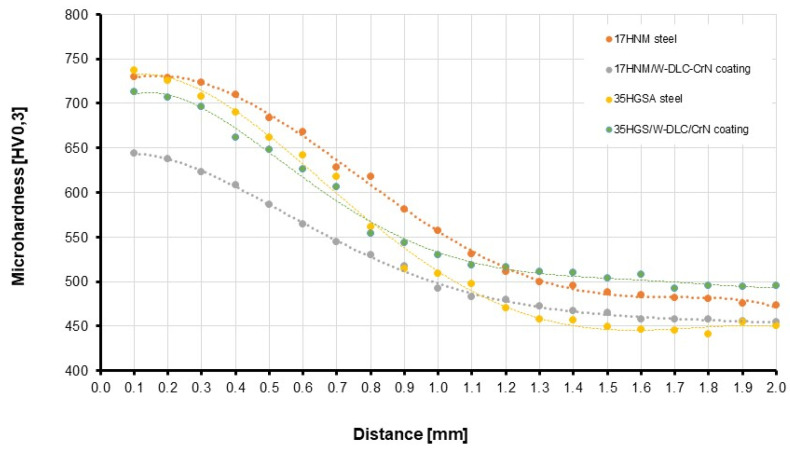
Microhardness profiles.

Figure 4 shows microhardness profiles obtained using a Future-Tech Corp FM-800 microhardness tester (Tokyo, Japan). The hardness curve for the 35HGSA steel with a coating shows an increase in core hardness to 550 HV, compared to the hardness curve for the 35HGSA steel without a coating, where hardness was 450 HV. As the main objective of the study was to increase the scuffing resistance of the working surface of the teeth after coating deposition, this is very advantageous. Thus, the selection of the 35HGSA steel for scuffing tests was deemed correct. On the contrary, for the 17HNM steel, microhardness decreased due to phase transformations (tempering) during the coating deposition process.

**Figure 5:**
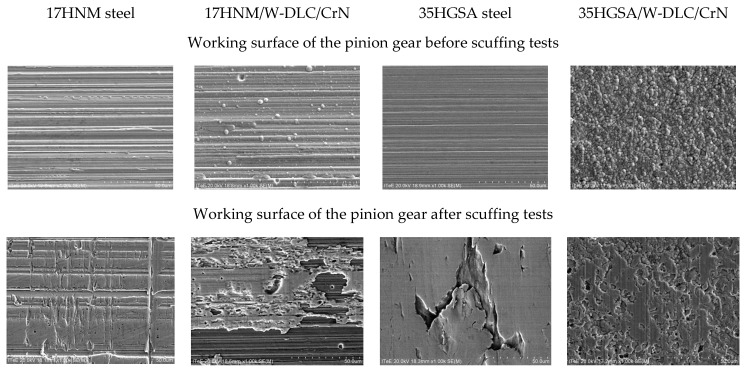
Results of working surface tests obtained using an SEM microscope.

Microscopic methods were used to observe the working surface of the pinion teeth. Figure 5 presents the results of observations employing a Hitachi SU-70 scanning electron microscope integrated with a Thermo Scientific NSS 312 (Madison, WI, USA) energy dispersive spectrometer (EDS). SEM images were taken for the working surface of the pinion teeth from an area of 100 µm × 100 µm. After scuffing tests, characteristic grooves and micropitting-related chipping were observed on the surface of the 17HNM and 35HGSA steel, respectively.

**Figure 6:**
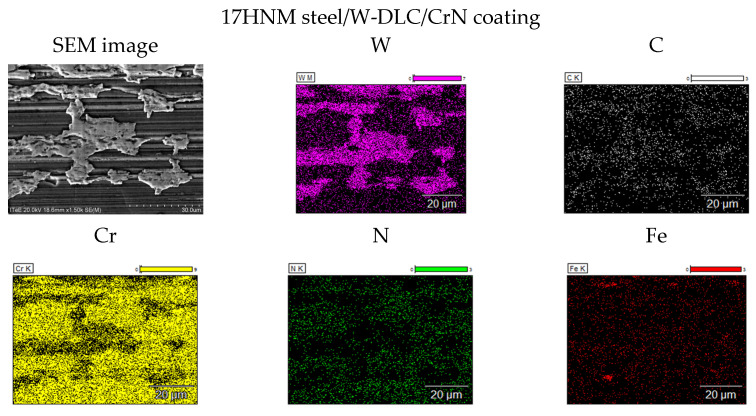

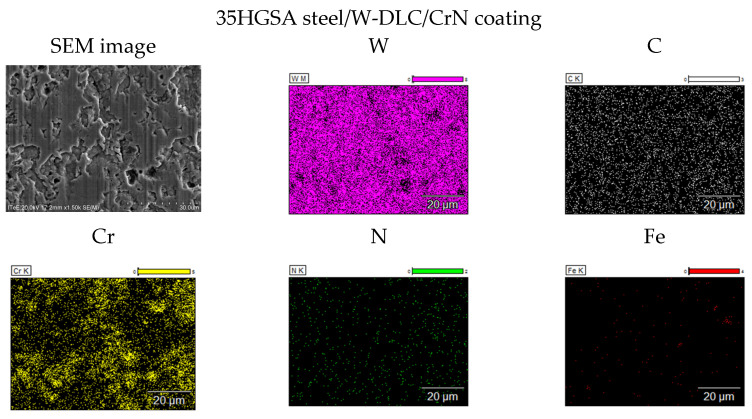
SEM images and maps of the EDS elemental distribution from the working surface of tooth. 17HNM/coating and 35HGSA/coating after scuffing tests at 1500× magnification.

The analysis of the EDS elemental distribution map (Figure 6) of the surface of a tooth made of the 17HNM steel with a W-DLC/CrN coating, after scuffing tests under severe conditions, showed that the W-DLC layer was worn through as deep as to the CrN interlayer. In contrast, the analysis of the surface of the tooth made of the 35HGSA/W-DLC-CrN steel did not show any coating wear. The presence of a given element on the tested surface is marked with colours. Magenta indicates the presence of tungsten, white indicates the presence of carbon, yellow indicates the presence of chromium, and green indicates the presence of nitrogen; these are the elements that make up the W-DLC/CrN coating. Red indicates the presence of iron, an element from the substrate. Its presence on the surface indicates that the coating has been rubbed off.

**Figure 7:**
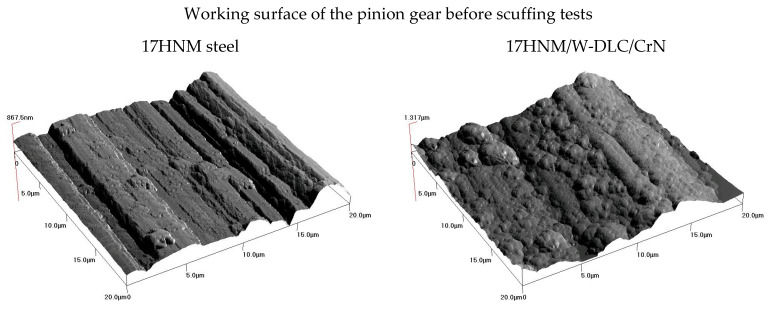

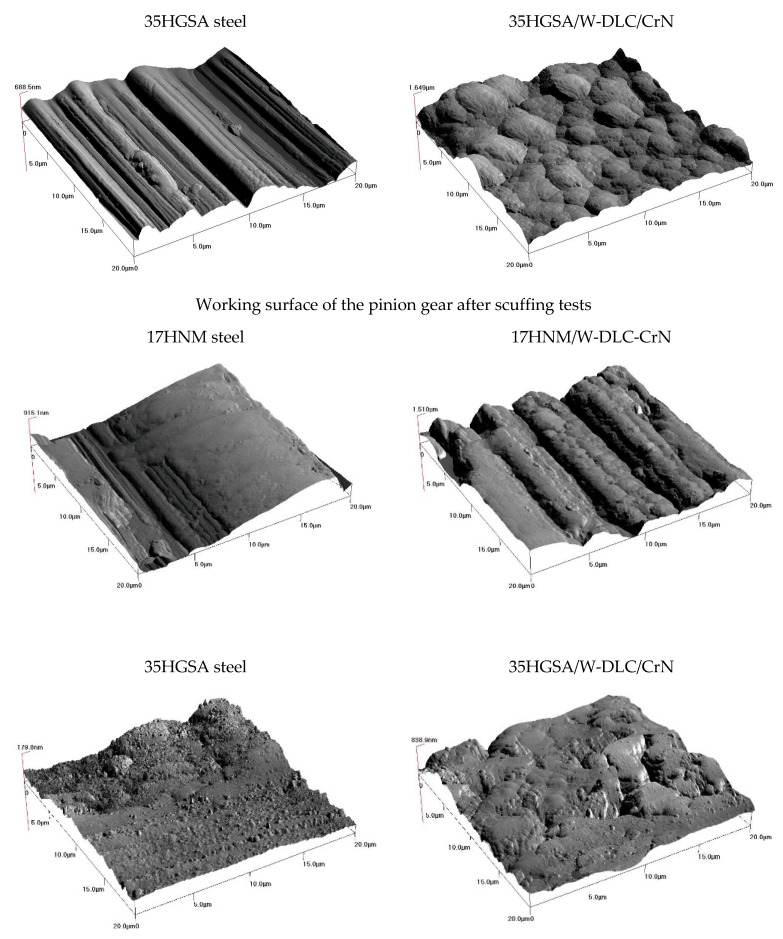
Results of working surface tests obtained using an AFM microscope.

Figure 7 presents 3D microscopic images of the working surfaces of teeth (20 µm × 20 µm) taken with a Quesant Instrument Corporation AFM Q-scope 250 atomic force microscope, which enabled the observation of surface details after scuffing tests. The nature of the changes observed on the examined surface confirmed the SEM analysis results. As a result of lapping, grinding marks were removed from the surface of the 17HNM steel tooth, and grooves appeared perpendicular to the grinding direction. The surface of the 35HGSA steel tooth showed significant destruction, with numerous indentations observed. The analysis of the surface of the 35HGSA/W-DLC/CrN steel tooth indicated the presence of the coating, while for the 17HNM/W-DLC-CrN steel tooth, the absence of the coating revealed the substrate surface after grinding.

**Figure 8:**
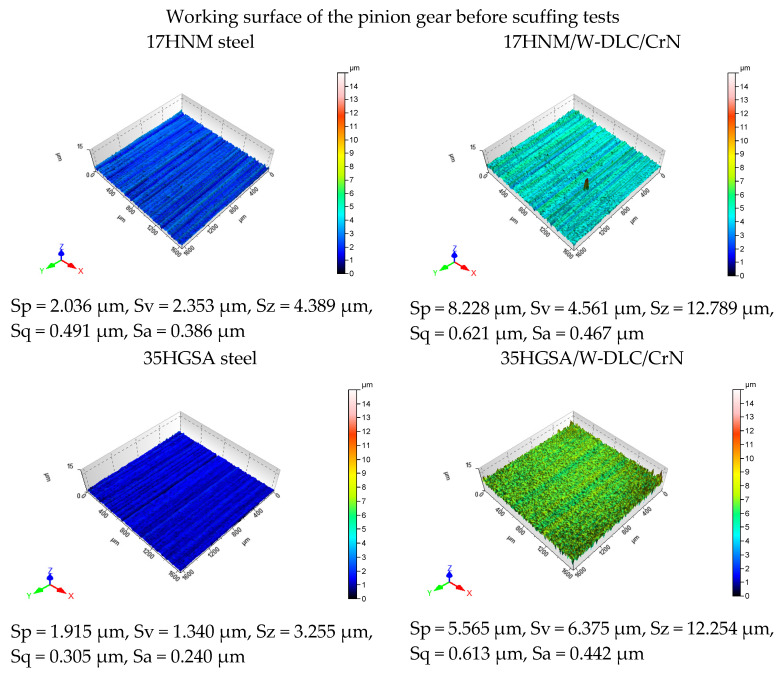
Results of pre-scuffing tests of the pinion’s working surface obtained using a WLI microscope.

**Figure 9:**
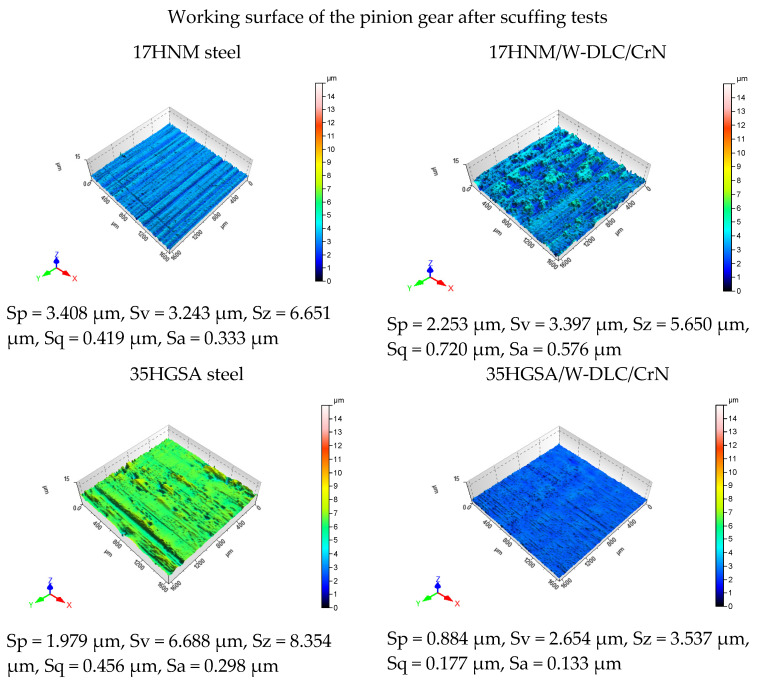
Results of post-scuffing tests of the pinion’s working surface obtained using a WLI microscope.

Figure 8 and Figure 9 show images of the working surfaces of the small gear teeth (1600 µm × 1600 µm) taken with a WLI interferometric microscope for 3D surface imaging at micro scale. Figure 8 presents 3D images of the working surface of the pinion before scuffing tests, while Figure 9 shows 3D images of the working surface after scuffing tests. The condition of the measured surface area is described by the following parameters: Sp—maximum peak height; Sv—maximum indentation depth; Sz—maximum surface height; Sa—arithmetic mean deviation of the surface roughness; and Sq—mean square deviation of the surface roughness. The use of interferometric microscopy not only allowed for a spatial representation of the surface, but also enabled a quantitative analysis of the characteristics of the examined area. The analysis of the roughness parameter values showed that the surface of the 35HGSA/W-DLC/CrN steel tooth after scuffing tests had the lowest values of the Sa (0.133 µm) and Sq (0.177 µm) parameters. On the other hand, the highest values of the Sv (6.688 µm) and Sz (8.354 µm) parameters were observed for the 35HGSA steel, which, compared to the surface before tribological tests, indicates surface destruction.

The images taken with optical, scanning electron, and atomic force microscopes enabled a qualitative analysis of the surface studied.

## 4. Conclusions

The results of the studies on the working surface of the pinion tooth allowed for the following conclusions:

- Gears made of nanobainitized 35HGSA steel, after W-DLC/CrN coating deposition, achieved scuffing resistance at load level 10;
- The W-DLC/CrN coating deposited on the surface of gears made of the 35HGSA steel was not worn through;
- The working surface of the tooth made of the 35HGSA steel without the coating was subjected to scuffing at load level 10; additionally, dangerous forms of micropitting wear were observed;
- The working surface of teeth made of the 17HNM steel, after the coating deposition process, was subjected to scuffing.

The aim of the scuffing tests was to demonstrate that the initially low scuffing resistance of the working surface of the pinion made of the 35HGSA steel increases after coating deposition at the highest failure load stage (FLS). Comparing the working surface of the pinion teeth made of both 17HNM and 35HGSA steels, with and without the W-DLC/CrN coating, the best performance was observed for the 35HGSA/W-DLC/CrN steel surface.

## Figures and Tables

**Table 1:** Properties of the W-DLC/CrN coating applied to 17HNM steel and 35HGSA steel.

Parameter	Substrate	
	17HNM Steel	35HGSA Steel
Thickness [um]	4.8	3.8
Adhesion [N]	40	50
Hardness [GPa]	13.0	10.0
Roughness Ra [um]	0.39	0.34
Roughness Sa [um]	0.47	0.44

**Table 2:** Modes of wear of the test pinion (small gear) and an example of pinion working surface polishing after scuffing tests.

Mode of Wear	Symbol	Appearance	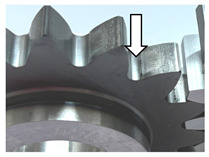
Polishing	W	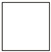
Scratches	R	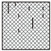
Scoring	B	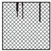
Scuffing	Z	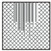

**Table 3:** Modes of the wear of the test pinion at particular load stages for the tested material combinations together with the total area of failures on the pinion (A_p_).

Load stage	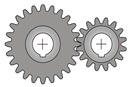 17HNM steel	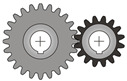 17HNM/W-DLC/CrN coating	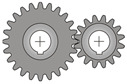 35HGSA steel	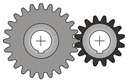 35HGSA/W-DLC/CrN coating
4	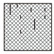 A_p_ ≈ 2.0 mm^2^	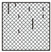 A_p_ ≈ 6.0 mm^2^	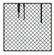 A_p_ ≈ 8.0 mm^2^	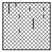 A_p_ ≈ 0.0 mm^2^
5	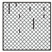 A_p_ ≈ 10.0 mm^2^	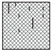 A_p_ ≈ 7.0 mm^2^	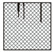 A_p_ ≈ 16.0 mm^2^	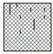 A_p_ ≈ 0.0 mm^2^
6	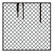 A_p_ ≈ 10.0 mm^2^	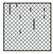 A_p_ ≈ 6.0 mm^2^	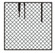 A_p_ ≈ 15.0 mm^2^	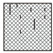 A_p_ ≈ 0.0 mm^2^
7	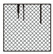 A_p_ ≈ 10.0 mm^2^	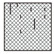 A_p_ ≈ 7.0 mm^2^	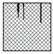 A_p_ ≈ 16.0 mm^2^	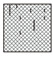 A_p_ ≈ 0.0 mm^2^
8	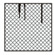 A_p_ ≈ 12.0 mm^2^	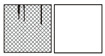 A_p_ ≈ 13.0 mm^2^	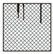 A_p_ ≈ 46.0 mm^2^	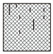 A_p_ ≈ 2.0 mm^2^
9	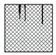 A_p_ ≈ 12.0 mm^2^	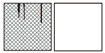 A_p_ ≈ 22.0 mm^2^	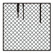 A_p_ ≈ 54.0 mm^2^	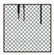 A_p_ ≈ 4.0 mm^2^
10	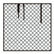 A_p_ ≈ 23.0 mm^2^	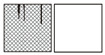 A_p_ ≈ 52.0 mm^2^	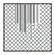 A_p_ ≈ 144.0 mm^2^	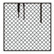 A_p_ ≈ 7.0 mm^2^

**Table 4:** Working surface of the pinion gear after scuffing tests under stringent conditions at the maximum failure load stage (FLS).

Pinion Material	The Working Surface of the Pinion Gear after Scuffing Tests			
	FLS	Test Gear	Working Surface of The Pinion Tooth	Optical Microscope Image
17HNM steel	>10	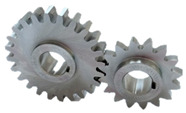	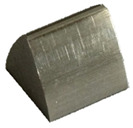	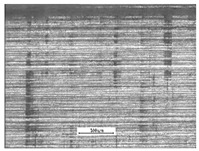
17HNM/ W-DLC/CrN coating	>10	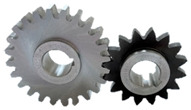	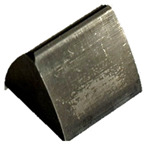	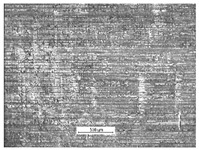
35HGSA steel	10	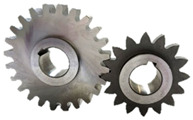	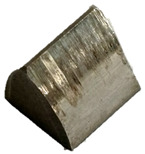	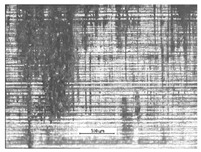
35HGSA/ W-DLC/CrN coating	>10	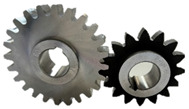	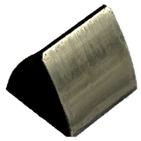	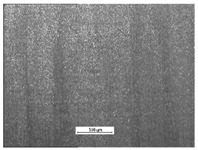

## Data Availability

The data presented in this study are available on request from the corresponding author. The data is not publicly available due to the fact that information regarding the transmission technology is reserved by the manufacturer.

## Abbreviations

*FLS*	failure load stage
*FZG*	the Gear Research Centre (Forschungsstelle fur Zahnrader und Getriebebau, FZG) of the Technical University of Munich
*PACVD*	Plasma Assisted Chemical Vapor Deposition
*GDOES*	Glow Discharge Optical Emission Spectrometer
*SEM*	Scanning Electron Microscope
*EDS*	Energy Dispersive Spectrometer
*WLI*	White Light Interferometer Microscope
*AFM*	Atomic Force Microscope
*Ra*	profile roughness [µm]
*Sa*	surface roughness [µm]
*Sp*	maximum peak height [µm]
*Sv*	maximum indentation depth [µm]
*Sz*	maximum surface height [µm]
*Sq*	mean square deviation of the surface roughness [µm]
*Ap*	wear surface area [mm^2^]
